# A Study of the Relationship Between Mental Health and Online Platforms for Education

**DOI:** 10.1192/bjo.2025.10159

**Published:** 2025-06-20

**Authors:** Nastaran Kazemi Rad, Atefeh Mohammad Jafari, Zahra Mirsepassi, Pedram Sarikhani

**Affiliations:** Tehran University of Medical Sciences, Tehran, Iran, Islamic Republic of

## Abstract

**Aims:** 1. Providing practical solutions for richer and broader use of online platforms for education among students. 2. Finding factors related to online education that are involved in causing mental disorders in students and preventing and correcting them.

**Methods:** This cross-sectional study was conducted after obtaining approval from the Research Council and Ethics Committee of Tehran University of Medical Sciences. Medical students were selected through simple random sampling, and those who met the inclusion criteria completed an online questionnaire that included the PHQ-9 and PSS-10 questionnaires developed by the researcher. Informed consent was obtained from all participants, and the data was analysed using SPSS software.

**Results:** A total of 330 medical students participated in the study, of whom 137 (41.5%) were female and 193 (58.5%) were male. The results revealed that 73.5% of participants had mild depression, 17.3% had moderate depression, and 9.4% had severe depression. Additionally, 4.2% had mild stress, 91.5% had moderate stress, and 4.2% had severe stress.

**Conclusion:** The study findings revealed that virtual education quality during the Covid-19 era, accessibility to virtual learning resources, financial and health worries due to Covid-19, and communication level among students with their family and friends significantly influence their mental health. The study suggests that while designing virtual classrooms, it is essential to consider the availability of resources, facilitate peer-group communication, and provide financial support to enhance students’ mental health.

